# Toll-like receptor 2 (TLR2) induces migration and invasive mechanisms in rheumatoid arthritis

**DOI:** 10.1186/s13075-015-0664-8

**Published:** 2015-06-09

**Authors:** Trudy McGarry, Douglas J. Veale, Wei Gao, Carl Orr, Ursula Fearon, Mary Connolly

**Affiliations:** Department of Rheumatology, St. Vincent’s University Hospital, Elm Park, Dublin Academic Health Care and The Conway Institute of Biomolecular and Biomedical Research, University College Dublin, Belfield, Dublin 4, Ireland

## Abstract

**Introduction:**

This study investigates the role of Toll-like receptor 2 (TLR2) in the regulation of migratory and invasive mechanisms in rheumatoid arthritis (RA).

**Methods:**

Invasion, migration, matrix metalloproteinase (MMP)-1, -3 and tissue inhibitor of matrix metalloproteinase-3 (TIMP-3) expression, β-integrin binding, cytoskeletal rearrangement and Ras-related C3 botulinum toxin substrate 1 (Rac1) activation in response to a TLR2-ligand, Pam3CSK4 (1 μg/ml), in ex vivo RA synovial tissue explants, primary RA synovial fibroblasts (RASFC) and microvascular endothelial cells (HMVEC) were assessed by Transwell Matrigel™ invasion chambers, enzyme-linked immunosorbent assay (ELISA), multiplex adhesion binding assay, reverse transcription polymerase chain reaction (RT-PCR), F-actin immunofluorescent staining, matrigel synovial outgrowths, Rac1 pull-down assays/Western blot and zymography. β1-integrin expression in RA/control synovial tissue was assessed by immunohistology. The effect of Pam3CSK4 on cell migration, invasion, MMP-3 and Rac1 activation was examined in the presence or absence of anti-β1-integrin (10 μg/ml) or anti-IgG control (10 μg/ml). The effect of an anti-TLR-2 mAb (OPN301)(1 μg/ml) or immunoglobulin G (IgG) control (1 μg/ml) on RASFC migration and RA synovial tissue MMP activity was assessed by wound assays, ELISA and zymography.

**Results:**

Pam3CSK4 significantly induced cell migration, invasion, MMP-1, MMP-3, MMP-2 and MMP-9 expression and induced the MMP-1/TIMP-3 and MMP-3/TIMP-3 ratio in RASFC and explants (*p* <0.05). β1-integrin expression was significantly higher in RA synovial tissue compared to controls (*p* <0.05). Pam3CSK4 specifically induced β1-integrin binding in RASFC (*p* <0.05), with no effect observed for β2-4, β6, αvβ5 or α5β1. Pam3CSK4 increased β1-integrin mRNA expression, Rac1 activation, RASFC outgrowths and altered cytoskeletal dynamic through induction of filopodia formation. Pam3CSK4-regulated cell migration and invasion processes, but not MMP-3, were inhibited in the presence of anti-β1-integrin (*p* <0.05), with no effect observed for anti-IgG control. Furthermore, anti-β1-integrin inhibited Pam3CSK4-induced Rac1 activation. Finally, blockade of TLR2 with OPN301 significantly decreased spontaneous release of MMP-3, MMP-2 and MMP-9 and increased TIMP-3 secretion from RA synovial explant cultures (*p* <0.05). Incubation of RASFC with OPN301 RA ex vivo conditioned media inhibited migration and invasion compared to IgG control.

**Conclusions:**

TLR2 activation induces migrational and invasive mechanisms, which are critically involved in the pathogenesis of RA, suggesting TLR2 as a potential therapeutic target for the treatment of RA.

**Electronic supplementary material:**

The online version of this article (doi:10.1186/s13075-015-0664-8) contains supplementary material, which is available to authorized users.

## Introduction

Rheumatoid arthritis (RA) is a chronic progressive autoimmune disease, characterised by synovial proliferation, neovascularisation and leucocyte extravasation, joint destruction and functional disability. RA synoviocytes manifest an abnormal phenotype with increased proliferation, resistance to apoptosis and invasiveness of adjacent tissue [1–3]. This results in synoviocyte hyperplasia, which transforms the synovial membrane (SM) into an aggressive, tumour-like tissue ‘pannus’, which is capable of destroying adjacent articular cartilage and bone.

Toll-like receptors (TLRs) have been implicated in the pathogenesis of RA with studies showing increased TLR2 and TLR4 expression in the perivascular regions of the joint, [4] at the sites of attachment and invasion into cartilage/bone, and on synovial macrophages [5]. Bacterial peptidoglycan (PG), a TLR2 ligand, has been detected in RA synovial fluids [6]. Increased expression of TLR2 has been demonstrated in collagen-induced arthritis, and streptococcal cell wall (SCW)-induced arthritis does not develop in TLR2-deficient mice [7]. Furthermore, studies have demonstrated that dominant negative forms of the TLR2 adapter molecules myeloid differentiation primary response gene 88 (MyD88) and MAL/TIRAP inhibit pro-inflammatory cytokine production in RA synoviocytes [8]. Recent data has shown that the pattern-recognition receptor nucleotide-binding oligomerisation domain-containing protein 1 (NOD1) synergises with TLR2 in the induction of cytokines in RA synovial fibroblast cells (RASFC) [9]. TLR2-induced cytokine expression has also been shown to be mediated by miR-19 in RASFC [10]. Furthermore, studies have shown the involvement of the Tie-2 receptor in mediating TLR2-induced angiogenesis [11]. Together these studies suggest that TLR2 is a key mediator involved in promoting cell migrational, invasive and destructive mechanisms in the RA joint.

Cell migration is essential for tissue infiltration during the inflammatory process and is initiated by cell polarisation and the formation of membrane protrusions at the leading edge [12]. Integrin-linked activation of the small guanosine triphosphates (GTPases) of the Rho family (Cdc42, Rho and Rac1) mediates cytoskeletal dynamics such as filopodia and lamellipodia formation and stress fibre formation, which is essential for cell migration [13, 14]. Integrin-linked activation of Rho GTPases mediates gene transcription, cell cycle progression and adhesion [13, 15]. Previous studies have demonstrated that integrin blockade can inhibit fibroblast invasion and adhesion of cells to the extracellular matrix (ECM) [16, 17]. In particular, blockade of α2β1 integrin in RA protects against cartilage destruction via reduction of matrix metalloproteinase (MMP)-3 [18]. In this study we investigate the effect of TLR2 on migrational and invasive mechanisms in RA. We demonstrate that activation of TLR2 induces RASFC migration, invasion and induction of MMPs. Furthermore, we show that TLR2-induced migration/invasion is partly mediated by β1 integrin and downstream alterations in cytoskeletal dynamics and Rac1 activation. Finally, using an ex vivo RA synovial explant model and RASFC, we demonstrated that an anti-TLR2 antibody (OPN301) significantly inhibited MMP expression and migration. Together this study further demonstrates that TLR2 activation plays a key role in regulating invasive mechanisms in RA, a key process involved in the pathogenesis of RA.

## Materials and methods

### Patients and RA synovial tissue

Patients with RA were recruited from the outpatient clinics at the Department of Rheumatology, St. Vincent’s University Hospital (SVUH). Only patients with an actively inflamed knee joint and fulfilling the revised American College of Rheumatology criteria were recruited [19]. RA synovial tissue was obtained at arthroscopy under direct visualisation as previously described [20]. Normal synovial tissue was obtained from ten patients (eight men; two women) undergoing interventional arthroscopy for cruciate ligament tears. Fully informed written consent was obtained from each patient prior to inclusion. This study was approved by the SVUH Ethics and Medical Research Committee.

### Immunohistochemistry

Synovial biopsies obtained at arthroscopy were snap frozen in OCT compound and stored at −80 °C. Seven-micron-thick sections were cut using a cryostat and placed on glass slides coated with 2 % 3-amino-propyl-triethoxy-silane (Sigma-Aldrich Ireland Ltd, Dublin, Ireland), dried overnight at room temperature and stored at −80 °C. To examine β1-integrin expression and localised distribution in the synovium, immunohistochemical analysis was performed in RA (*n* = 17) and normal synovial tissue (*n* = 9). Tissue sections were fixed in acetone for 10 min and air-dried. Non-specific binding was blocked using 10 % casein buffer. A routine three-stage immunoperoxidase labelling technique incorporating avidin-biotin-immunoperoxidase complex (Dako, Glostrup, Denmark) was used. Sections were incubated with mouse polyclonal anti-β1-integrin (R&D Systems, Cambridge, UK) at room temperature for 1 h. Sections were also incubated with an appropriate isotype-matched mouse-immunoglobulin G (IgG) control antibody (R&D Systems, Cambridge, UK) as negative controls. Colour was developed in solution containing diaminobenzadine-tetrahydrochloride (Sigma-Aldrich), 0.5 % H_2_O_2_ in phosphate-buffered saline (PBS) buffer (pH 7.6). Slides were counterstained with haematoxylin (BDH Laboratory Supplies, Poole, UK) and hydration and fixation was performed through a series of IMS and xylene solutions. Sections were mounted using DPX mountant (BDH Laboratory Supplies). Images were captured using Olympus DP50 light microscope and analysis software (Soft Imaging System Corporation, Lakewood, CO, USA). Slides were analysed using a well-established semi-quantitative scoring method (0–4) and scored separately for perivascular/vascular, lining layer and sub-lining layer regions [21].

### Ex vivo synovial explants

RA synovial biopsies taken at the time of arthroscopies were sectioned into two equal pieces and cultured in 96-well plates in full RPMI medium. This culture models maintains the synovial architecture and cell–cell contact, and therefore more closely reflects the in vivo environment. For stimulation experiments, RA synovial biopsies were cultured with Pam3CSK4 (1 μg/ml) or basal control media for 24 h. For blockade experiments, biopsies were cultured in the presence of OPN301 (mouse IgG1 monoclonal anti-TLR2 antibody, a kind gift from Opsona Therapeutics, Dublin, Ireland, 1 μg/ml) or IgG isotype control (mouse IgG1 isotype control, Opsona Therapeutics; 1 μg/ml). For each experiment, three technical replicates are combined (three individual biopsies from one patient) to give one biological sample, and this was performed on a number of different patients. This is important to allow for heterogeneity within the patient joint and between patients. Following incubation, culture supernatants were harvested and assayed for MMPs by enzyme-linked immunosorbent assay (ELISA) and zymography. For further functional experiments, 10 % conditioned media from OPN301 or IgG1-treated ex vivo synovial tissue was co-cultured with primary RASFC and invasion, migration and MMP expression was assessed as outlined below.

### Isolation and culture of primary RASFC

RA synovial biopsies were digested with 1 mg/ml collagenase type 1 (Worthington Biochemical Corp., Freehold, NJ, USA) in RPMI medium (Gibco-BRL, Paisley, UK) for 4 h at 37 °C in humidified air with 5 % CO_2._ Dissociated cells were grown to confluence in RPMI 1640 medium, 10 % fetal calf serum (FCS) (Gibco-BRL), 10 ml of 1 mmol/l HEPES (Gibco-BRL), penicillin (100 units/ml; Bio-sciences Ltd., Dublin, Ireland), streptomycin (100 units/ml; Bio-sciences) and fungizone (0.25 μg/ml; Bio-sciences) before passaging.

### Culture of HMVEC

Human microvascular endothelial cells (HMVEC) (Lonza, Walkersville, MD, USA) were incubated in endothelial cell basal medium (EBM: Lonza) supplemented with endothelial cell growth medium (EGM) microvascular bullet kit containing 25 ml FCS, 0.5 ml hEGF, 0.5 ml hydrocortisone, 0.5 ml gentamicin, 0.5 ml bovine brain extract (Lonza). Cells were grown to confluence in T75cm flasks at 37 °C in humidified air with 5 % CO_2_ prior to being harvested with trypsin-EDTA (Lonza). HMVEC between the fourth and eight passages were used for experiments.

### Ex vivo RA fibroblast outgrowths

To examine RA synovial outgrowths, an ex vivo synovial tissue explant model was utilised [22]. A total of 50 μl of matrigel (Bio-sciences) was added to each well of a 96-well plate and incubated for 1 h at 37 °C. Following this, RA synovial tissue was sectioned and placed in matrigel wells with RPMI 1640 medium supplemented with 10 % FCS (Gibco-BRL), 10 ml of 1 mmol/l HEPES (Gibco-BRL), penicillin (100 units/ml; Bio-sciences), streptomycin (100 units/ml; Bio-sciences) and fungizone (0.25 μg/ml; Bio-sciences). RA explants were stimulated with TLR2 ligand Pam3CSK4 (1 μg/ml) over a time course of 1–15days. Supernatants were collected every 4 days and replenished with fresh media and experimental agents. Images were taken using a phase-contrast microscope (a Nikon TMS microscope (Nikon Corp., Tokyo, Japan) linked to a Canon S70 camera (Canon Inc., Tokyo, Japan)).

### Immunofluorescent staining for F-actin

Primary RASFC were seeded sparsely into 8-well chamber slides and serum starved for 24 h and subsequently cultured with Pam3CSK4 (1 μg/ml) for a further 24 h. Slides were washed with PBS and fixed with 1 % paraformaldehyde in PBS for 20 min. To visualise F-actin, slides were stained with Oregon Green® phalloidin (Molecular Probes, Leiden, The Netherlands). Nuclei were counterstained with DAPI (Sigma-Aldrich, St. Louis, MO, USA). Stained cells were visualised with a Leitz DM40 microscope (Leica Microsystems, Wetzlar, Germany) and images were captured using the AxioCam system and AxioVision 3.0.6. software (Carl Zeiss Inc., Thornwood, NY, USA).

### β integrin-mediated cell adhesion array

The Chemicon Integrin-Mediated Cell Adhesion Array Kit (Chemicon International Inc., Temicula, CA, USA) utilises monoclonal antibodies generated against human integrins, which are immobilised onto a goat anti-mouse antibody-coated microplate and used to capture cells expressing these integrins on their cell surface. RASFC were grown to confluence, serum starved overnight and then cultured with Pam3CSK4 (1 μg/ml) for an additional 24 h. Single-cell suspensions were prepared using a non-enzymatic dissociation buffer (Sigma-Aldrich). A total of 100 μl (1 × 10^5^ cells in adhesion buffer) was added to each well of the Beta Integrin-Mediated Cell Adhesion Array Kit (Chemicon). After incubation for 2 h at 37 °C/5 % CO_2_, wells were washed and stained, and cell-bound stain was solubilised in extraction buffer. Integrin expression was determined by measuring the absorbance at 570 nm.

### Wound repair assays

RASFC and HMVEC were seeded onto 48-well plates and grown to confluence. A single scratch wound was induced through the middle of each well with a sterile pipette tip. Cells were subsequently stimulated for 24 h with Pam3CSK (1 μg/ml). RASFC and HMVEC migration across the wound margins from 12 to 24 h was assessed and photographed using a phase-contrast microscope (Nikon TMS microscope linked to a Canon S70 camera). To assess if the β1-integrin signalling mediates Pam3CSK4-induced cell migration, experiments were also performed in the presence or absence of anti-β1-integrin (10 μg/ml) or isotype-matched non-immune IgG (10 μg/ml). Semi-quantitative analysis of cell repopulation of the wound was assessed by two blinded observers as previously described [23]. Briefly, images of the scratch wound assays were taken at x20 magnification. The mean closure of the wound was manually calculated from the average of three individual measurements from each wound. This process was repeated for all technical replicates. Measurement of scratches at time 0 were designated as 100 % open. From this, the percentage of closure for all scratches was calculated. Twenty-four percent wound closure represents the distance the cells migrated into the scratch wound. Data is represented as mean ± SEM. Furthermore to assess the effect of blocking TLR2 on cell migration, RASFC were incubated with 10 % conditioned media from ex vivo RA synovial explants cultured either with OPN301 (1 μg/ml) or IgG isotype control (1 μg/ml) for 24 h.

### Transwell migration assay

Biocoat Matrigel™ Invasion Chambers (Becton Dickinson, Oxford, UK) were used to assess RASFC and HMVEC invasion. Cells were seeded at a density of either 2.5 × 10^4^ cells per well in the migration chamber on 8 μm membranes pre-coated with matrigel. Cells were incubated for either 24 h (HMVEC) or 48 h (RASFC). Initial experiments assessed the effect of Pam3CSK4 (1 μg/ml) on HMVEC and RASFC invasion. To assess if the β1-integrin signalling pathways mediate Pam3CSK4-induced cell invasion, experiments were also performed in the presence or absence of anti-β1-integrin (10 μg/ml) or isotype-matched non-immune IgG (10 μg/ml). To determine if TLR2 blockade could further inhibit invasion, RASFC were cultured in the presence of 10 % RA ex vivo explant-conditioned media previously cultured with either anti-TLR2 antibody OPN301(1 μg/ml) or IgG control (1 μg/ml). Non-migrating RASFC and HMVEC were removed from the upper surface by gentle scrubbing. Migrating cells attached to the lower membrane were fixed with 1 % glutaraldehyde and stained with 0.1 % crystal violet. Cells from five random high-power fields for each well were counted to assess the average number of migrating cells [24].

### Rac1 pull-down assay

Rac1 activity was determined using an Active Rac1 Pull-Down Detection Kit (Thermo Scientific, Pierce, Rockford, IL, USA) as per the manufacturer’s protocol. RASFC were stimulated with Pam3CSK4 (1 μg/ml) for 12 h. After treatment, cells were rinsed once with ice-cold PBS and scraped into 500 μl of lysis buffer. Samples were vortexed and centrifuged at 16,000 g for 15 min at 4 °C and the supernatant was transferred to a new tube. Active Rac1 was then affinity purified with GST-Pak-PBD (Pak 1-p21-binding domain), separated by SDS-PAGE and measured by Western blotting using anti-Rac1 antibodies and total Rac1 protein. To assess if the β1-integrin signalling pathways mediate Pam3CSK4-induced Rac1 activation, experiments were also performed in the presence or absence of anti-β1-integrin (10 μg/ml) or isotype-matched non-immune IgG (10 μg/ml). The signal intensity of the appropriate bands on the autoradiogram was calculated using the Kodak EDAS 120 System (Kodak, Rochester, NY, USA). Densitometry analysis performed out using ImageJ software. Data is expressed as fold change compared to β-actin control.

### Matrix metalloproteinase expression

RASFC were seeded in 48-well plates containing RPMI plus supplements for 24 h. Following 24 h of incubation in serum-free RPMI 1640, cells were stimulated with Pam3CSK4 (1 μg/ml), experiments were also performed in the presence or absence of anti-β1-integrin (10 μg/ml) or isotype-matched non-immune IgG (10 μg/ml). For inhibitory experiments, RA ex vivo biopsies were treated as outlined above. Supernatants were harvested and levels of MMP-3, MMP-1 and tissue inhibitor of matrix metalloproteinase (TIMP)-3 were measured by specific ELISA (R&D Systems) according to the manufacturer’s conditions. The ELISA standard ranges were 10 ng/ml to 0.156 ng/ml for MMP-3 and MMP-1 and 4,000 pg/ml to 62.5 pg/ml for TIMP-3.

### Gelatin zymography

The activity of MMP-2 and MMP-9 secreted by ex vivo RA explants and RASFC was determined by gelatin zymography [25]. Following incubation, 10 μl supernatant was loaded into appropriate gel lanes. Zymogram gels consist of 7.5 % polyacrylaminde gels polymerised together with gelatin (1 mg/ml). Following electrophoresis, gels were washed with 2.5 % Triton X-100 and incubated with substrate buffer (50 mM Tris, 5 mM CaCl_2_, pH 7.5) at 37C for 24 h. Gels were then stained with Coolmassie brilliant blue R250 and destained with water. Bands were identified using gelatinase standards (Merck Millipore, Billerica, MA, USA).

### mRNA extraction and PCR analysis

RASFC were grown to confluence, cultured in serum-free RPMI medium for 24 h and then stimulated with Pam3CSK4 (1 μg/ml) for a further 24 h. Experiments were also performed in the presence or absence of anti-β1-integrin (10 μg/ml) or isotype-matched non-immune IgG (10 μg/ml). Total RNA was isolated using RNeasy Mini Kit (Qiagen, Venlo, The Netherlands) according to the manufacturer’s specifications. Purity of RNA was measured and samples with a ratio over 1.8 (260:280 nm) were used in subsequent experiments. Total RNA (200 ng) was reverse transcribed to cDNA. Relative quantification of gene expression was analysed with pre-optimised conditions using Lightcycler-480 PCR technology (Roche Diagnostics, Lewes, UK). Specific primers for *β1-integrin* (Hs00559595_m1) were used and primers for *18S* (Hs99999901_sl) ribosomal RNA were used as an endogenous control (Applied Biosystems, Carlsbad, CA, USA).

### Statistical analysis

SPSS 15.0 system (SPSS Inc, Chicago, IL, USA) for Windows was used for statistical analysis. Wilcoxon signed-rank test or Mann-Whitney was used for analysis of non-parametric data. Student *t* test was used for parametric data. *p* values of less than 0.05 (^*^p <0.05) were determined as statistically significant. All data is represented as mean ± SEM.

## Results

### TLR2 induces cell migration in RASFC and HMVEC

**Fig. 1:**
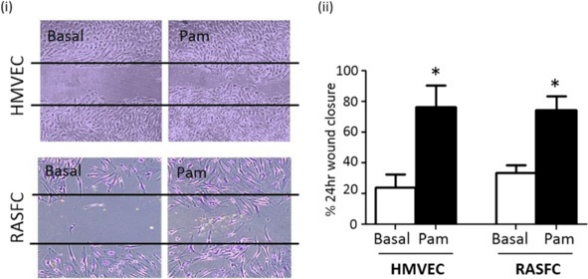
Representative photomicrograph demonstrating cells repopulating the wound in response to Pam3CSK4 (1 μg/ml) in RASFC and HMVEC (**i**). Bar graph quantifying RASFC and HMVEC (both *n* = 4) 24 h migration in response to Pam3CSK4 (**ii**). Data is represented as mean ± SEM, ^*^ *p* <0.05, significantly different to control. *HMVEC* human microvascular endothelial cells, *RASFC* rheumatoid arthritis synovial fibroblast cells

RASFC and HMVEC monolayers were wounded and stimulated in the presence or absence of Pam3CSK4 (1 μg/ml). Figure 1i shows representative images of wound repair in response to Pam3CSK4 for RASFC and HMVEC, where repopulation of wound margins was observed. Semi-quantitative analysis demonstrated a significant increase in cell migration in response to Pam3CSK4 (Fig. 1ii).

### TLR2 induces cell invasion in RASFC and HMVEC

**Fig. 2:**
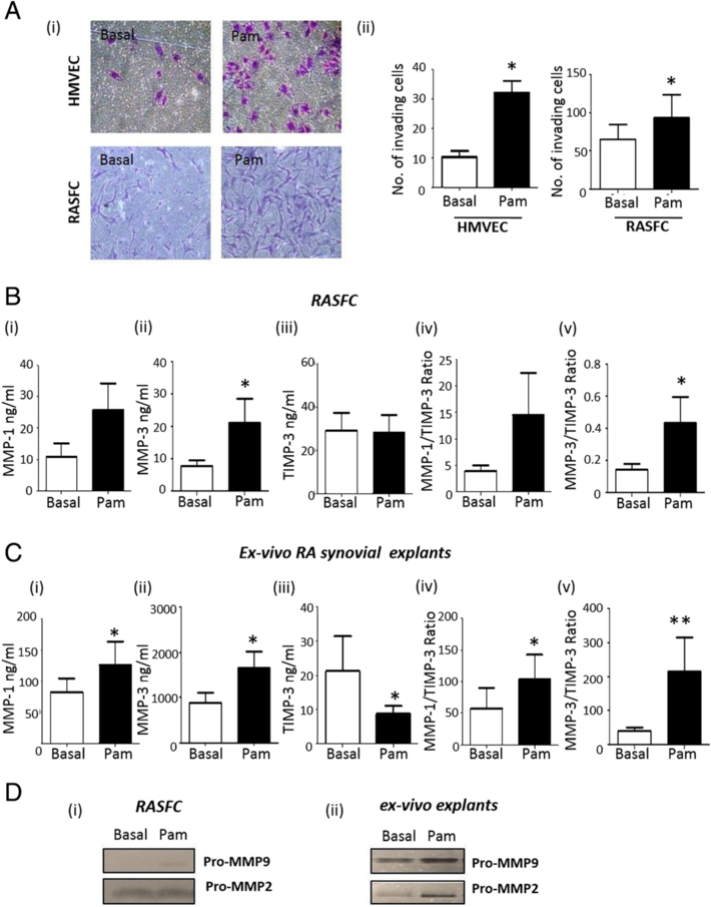
**a** (*i*) Representative images of Pam3CSK4 induces HMVEC and RASFC invasion compared to basal control. Following 24 h stimulation, invading cells attached to lower membrane were fixed (1 % glutaraldehyde) and stained (0.1 % crystal violet) (mag x40). (*ii*) Quantification of HMVEC (*n* = 4) and RASFC (*n* = 6) invasion. Quantification of RASFC (**b**) and RA synovial explant (**c**) MMP-1 (*i*), MMP-3 (*ii*) and TIMP-3 (*iii*) secretion and MMP-1/TIMP-3 (*iv*) and MMP-3/TIMP-3 ratio (*v*) following Pam3CSK4 stimulation (*n* = 7). **d** shows representative zymogram for pro-MMP-2 and pro-MMP-9 production in RASFC (*i*) and RA synovial tissue (*ii*) following 24 h stimulation with Pam3CSK4 (1 μg/ml) (*n* = 3). Data is represented as mean ± SEM, ^*^ *p* <0.05, ^**^ *p* <0.01, significantly different to control. *HMVEC* human microvascular endothelial cells, *MMP* matrix metalloproteinase, *RA* rheumatoid arthritis, *RASFC* rheumatoid arthritis synovial fibroblast cells, *TIMP* tissue inhibitor of metalloproteinase

To assess the effects of Pam3CSK4 on RASFC and HMVEC invasion, Transwell Matrigel™ invasion chambers were utilised. Representative images of increased RASFC and HMVEC invasion following Pam3CSK4 stimulation are shown in Fig. 2a (i). Quantitatively, RASFC and HMVEC invasion were significantly induced by Pam3CSK4 compared to control (*p* <0.05) (Fig. 2a (ii)). To further assess the invasive microenvironment, MMP-1, MMP-3 and TIMP-3 expression in RASFC and RA ex vivo synovial explants were measured by ELISA. Following Pam3CSK4 stimulation, MMP-1 expression increased 2.4-fold (basal 10.93 ng/mL compared to Pam3CSK4 25.76 ng/mL, *p* = 0.054) in RASFC and 1.6-fold in RA synovial tissue (basal 81 ng/mL compared to Pam3CSK4 126.3 ng/mL, *p* <0.05) (Fig. 2b, 2c (i)). MMP-3 expression increased 2.7-fold (basal 7.87 ng/mL compared to Pam3CSK4 21.16 ng/mL) in RASFC and 1.9-fold in RA synovial tissue (basal 895 ng/mL compared to Pam3CSK4 1675 ng/mL, *p* <0.05) (Fig. 2b, 2c (ii)). TIMP-3 secretion from RA ex vivo explants significantly decreased 2.4-fold (basal 21 ng/mL compared to Pam3CSK4 8.6 ng/mL, *p* <0.05) (Fig. 2c (iii)), with no significant change observed in RASFC (*p* = 0.09) (Fig. 2b (iii)). There was an increase in the ratio of MMPs to TIMP-3 in both RASFC and RA ex vivo explants. The MMP-1:TIMP-3 ratio was increased 3.7-fold in RASFC (3.9 to 14.5, *p* = 0.07) and 1.8-fold in RA ex vivo biopsies (57 to 103, *p* <0.05) (Fig. 2b, 2c (iv)). The MMP-3:TIMP-3 ratio also increased 2.9-fold in RASFC (0.15 to 0.44, *p* <0.05) and 5.2-fold in ex vivo RA synovial explants (41 to 215, *p* <0.01) (Fig. 2b, 2c (v)). Furthermore, MMP-9 was induced in both RASFC (Fig. 2d (i)) and RA synovial tissue (Fig. 2d (ii)) with an increase in MMP-2 observed in RA explants (Fig. 2d (ii)) following Pam3CSK4 activation as assessed by gelatin zymography.

### Pam3CSK4 induces ex vivo fibroblast outgrowth and cytoskeletal rearrangement

**Fig. 3:**
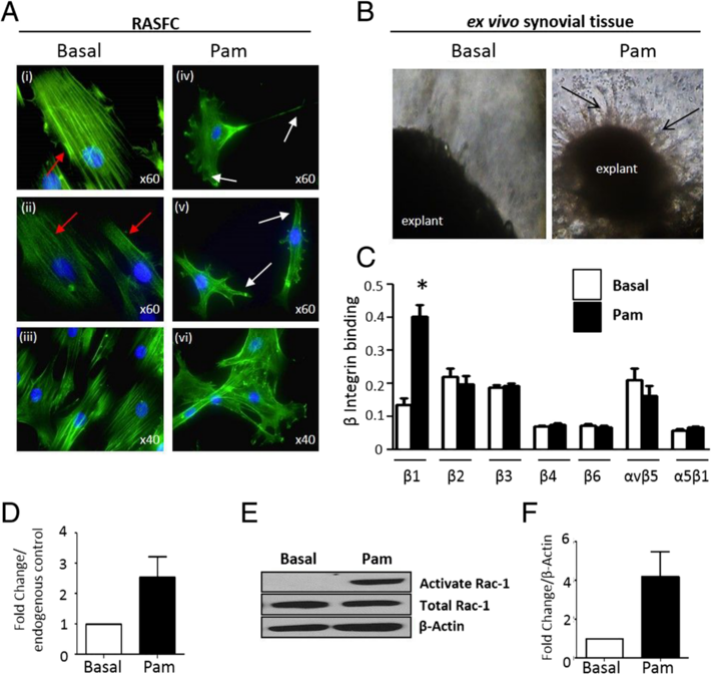
Representative photomicrograph showing cytoskeletal rearrangement of RASFC (**a**) and ex vivo synovial explant culture matrigel assays (**b**) in response to Pam3CSK4 (1 μg/ml). Pam3CSK4 induces cytoskeletal rearrangement in RASFC (*iv*–*vi*) compared to basal control (*i*–*iii*) as evidenced by loss of F-actin fibres (*green*) and formation of filopodial and lamellopodial protrusions (*red arrows*) (*n* = 3). Original magnification x60 (*i*, *ii*, *iv*, *v*) or x40 (*iii*, *vi*). (**b**) Pam3CSK4 (1 μg/ml) also induces synovial outgrowths from ex vivo RA explants as indicated by *black arrow* (*right panel*) compared to basal (*left panel*) (*n* = 3). **c** Bar graph quantifying beta integrin increased expression of β1-integrin on the cell surface of RASFC treated with Pam3CSK4 (1 μg/ml) (*n* = 5). **d** Bar graph representing β1-integrin mRNA in RASFC under basal conditions or stimulated with Pam3CSK4 (1 μg/ml) (*n* = 5). **e** Representative Western blot for activated Rac1, total Rac1 and β-actin control in RASFC following 24 h stimulation with Pam3CSK4 (1 μg/ml). **f** Densitometry quantification of active Rac1 in RASFC (*n* = 3). Data is represented as mean ± SEM, ^*^ *p* <0.05, significantly different to control. *RA* rheumatoid arthritis, *RASFC* rheumatoid arthritis synovial fibroblast cells

To further probe the effects of TLR2 activation on cell migration/invasion the effect of Pam3CSK4 on cytoskeletal dynamics was examined using F-actin staining. Figure 3a, in panels (i–iii), under basal or resting conditions, intact bundles of stress fibres are clearly visible and are orientated along the cells in a uni-directional manner. This is indicated in Fig. 3a (i) and (ii) by red arrows. Figure 3a panels (iv–vi) demonstrate the effects of Pam3CSK4 on the F-actin cytoskeletal architecture and cell shape. Pam3CSK4 has a dramatic effect on the architecture of the cell. It induces thin actin-rich extensions of the cytoplasm and membrane ruffling (observed in Fig. 3a (iv)), indicated by white arrows). It also induces filopodial and lamellipodial-like structures (observed in Fig. 3a (v)), indicated by white arrows). Lower magnification images (x40) depicted in Fig. 3a capture cells either under basal (iii) or Pam3CSK4-induced (vi) conditions, where these key cellular changes are uniform and highly noticeable amongst groups of cells. To further assess the role of TLR2, we cultured ex vivo RA whole tissue synovial explants embedded in matrigel for 15 days with Pam3CSK4 (1 μg/ml). Images of RA synovial outgrowths are shown in Fig. 3b where a significant increase in synovial outgrowth is demonstrated in response to Pam3CSK4 compared to basal control (indicated by black arrows) where minimal outgrowth was observed. Pam3CSK4 significantly increased expression of β1-integrin on the cell surface of RASFC (*p* <0.05) (Fig. 3c). No significant differences were observed in the levels of β2, β3, β4, β6, αVβ5 or α5β1 integrins. Figure 3d demonstrates a 2.6- ± 0.66 fold increase in β1-integrin mRNA following Pam3CSK4 stimulation. Furthermore Pam3CSK4 induced active Rac1 compared to basal control in RASFC, without change in global Rac1 expression (Fig. 3e). Figure 3f shows representative bar graph demonstrating a 4.2-fold increase in active Rac1 in response to Pam3CSK4.

### β1-integrin is expressed in RA synovial tissue

**Fig. 4:**
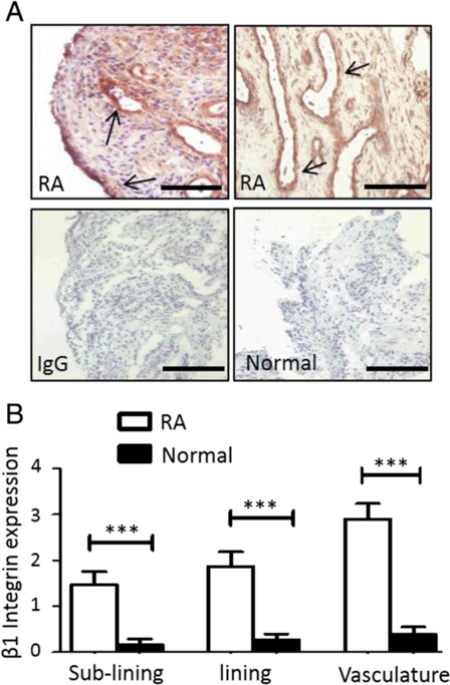
**a** Representative photomicrographs of immunohistochemical staining of β1-integrin expression in RA and normal synovial tissue. β1-integrin expression in RA synovial sections was localised to lining layer, sub-lining and vascular regions as indicated by *black arrows*. Representative images of β1-integrin minimal staining in healthy control (HC) tissue with no staining observed for IgG control. The scale bar (lower right-hand corner) represents a distance of 50 μm. **b** Representative bar graphs demonstrating increased β1-integrin expression in RA synovium, lining layer, sub-lining layer, peri-vasulature regions compared HC synovium. Data is represented as mean ± SEM, ^*^ *p* <0.05, ^**^ *p* <0.01, ^***^ *p* <0.005, significantly different to control. *IgG* immunoglobulin G, *RA* rheumatoid arthritis

Representative histological images demonstrate increased β1-integrin expression in RA synovial tissue compared to healthy control synovium (Fig. 4a). β1-integrin is highly expressed around the vasculature, in the synovial lining layer and also in the sub-lining layer of RA synovial tissue, as indicated by black arrows (Fig. 4a, upper panels). In contrast, β1-integrin expression in normal synovial tissue is minimal. No staining was observed for IgG control (Fig. 4a, lower left panel). Semi-quantification showed increased expression of β1-integrin in RA versus healthy control (HC) tissue for the perivascular/vascular regions (2.9 ± 0.34 vs 0.4 ± 0.16; *p* <0.005) sub-lining (1.5 ± 0.29 vs 0.3 ± 0.12, *p* <0.005), and lining layer (1.9 ± 0.32 vs 0.3 ± 012, *p* <0.005) (Fig. 4b).

### Pam3CSK4-induced cell migration and invasion is mediated through β1-integrin

**Fig. 5:**
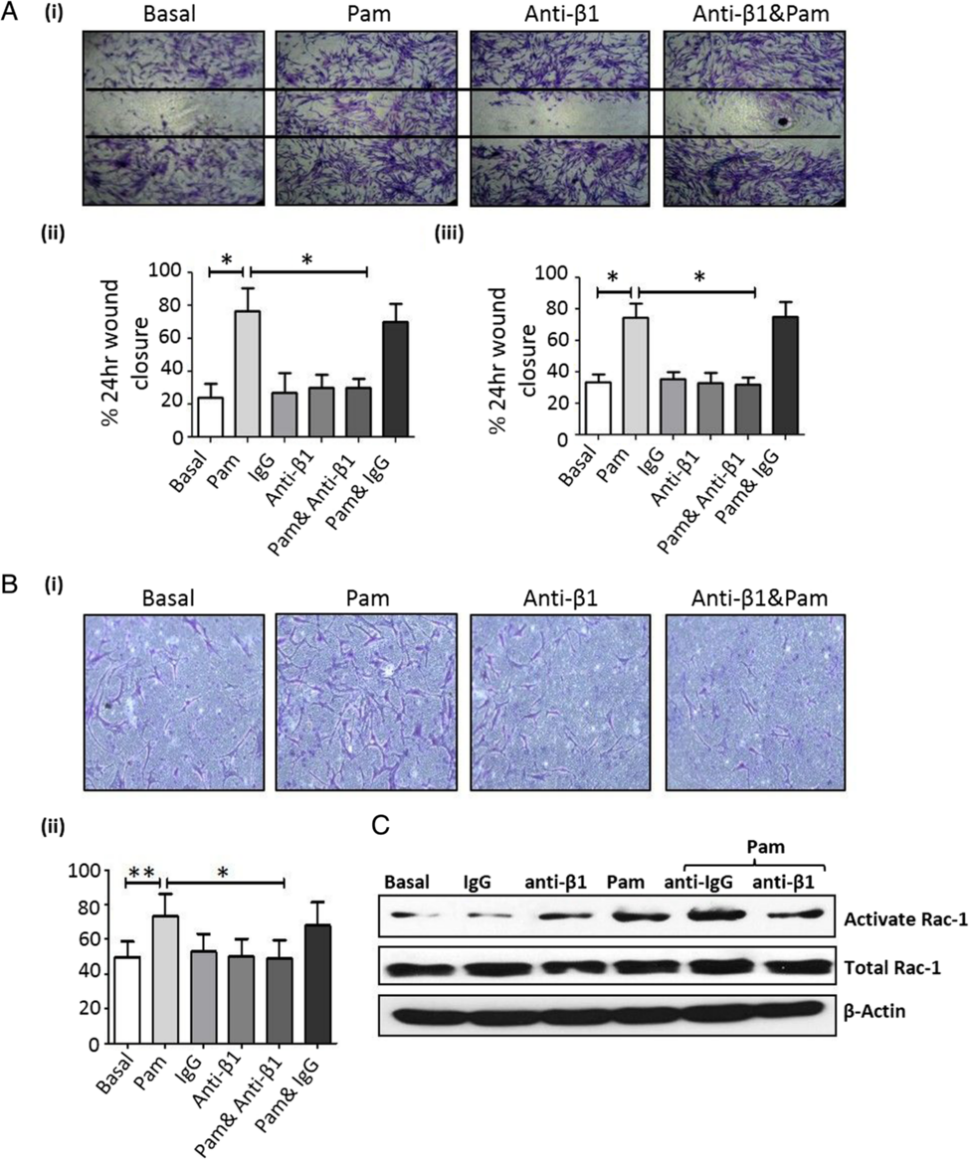
**a** Representative photomicrographs of RASFC inhibition of migration by anti-β1-integrin (10 μg/ml) are seen in (*i*). Bar graphs showing RASFC (*ii*) and HMVEC (*iii*) repopulating the wound in response to Pam3CSK4 (1 μg/ml), an effect that is inhibited by anti-β1-integrin (10 μg/ml) (*n* = 4). **b** Representative photomicrographs showing anti-β1-integrin (10 μg/ml) inhibits Pam3CSK4 (1 μg/ml)-induced RASFC invasion (i). Bar graph quantifying RASFC invasion (*ii*) (*n* = 4). **c** Western blot for Rac1, total Rac1 and β-actin control demonstrating that Pam3CSK4 (1 μg/ml)-induced Rac1 activation in RASFC is inhibited by the presence of anti-β1-integrin (10 μg/ml), with no effect observed for anti-IgG control monoclonal antibody (10 μg/ml). Total Rac1 was unchanged (n = 1) Data is represented as mean ± SEM, ^*^ *p* <0.05, ^**^ *p* <0.01, significantly different to control. *HMVEC* human microvascular endothelial cells, *IgG* immunoglobulin G, *RASFC* rheumatoid arthritis synovial fibroblast cells

To examine if Pam3CSK4-induced migration and invasion processes are in part mediated by the β1-integrin signalling pathways, RASFC and HMVEC were stimulated with Pam3CSK4 in the presence or absence of neutralizing antibody against β1-integrin or an isotype-matched anti-IgG control. Figure 5a demonstrates Pam3CSK4-induced RASFC and HMVEC migration was inhibited in the presence of anti-β1-integrin. Representative images of RASFC are shown in Fig. 5a (i). Semi-quantitative analysis demonstrated a significant decrease in Pam3CSK4-induced cell migration in response to anti-β1-integrin in both RASFC (Fig. 5a (ii)) and HMVEC (Fig. 5a (iii)) (*p* <0.05). Pam3CSK4-induced RASFC invasion was inhibited in the presence of anti-β1-integrin (*p* <0.05), with no effect observed for the IgG control (Fig. 5b (i, ii)). Similar effects were observed for HMVEC (Figure S1A in Additional file 1). Furthermore, Pam3CSK-induced RASFC Rac1 activation was inhibited by anti-β1-integrin in RASFC, with no change in global Rac1 expression (Fig. 5c; Figure S1B in Additional file 1). Interestingly, anti-β1-integrin had no effect on Pam3CSK4-induced MMP-3 (*p* = 0.87) (data not shown), suggesting that these were independent of β1-integrin.

### OPN301 inhibits migration and invasion RA explants and RASFC

**Fig. 6:**
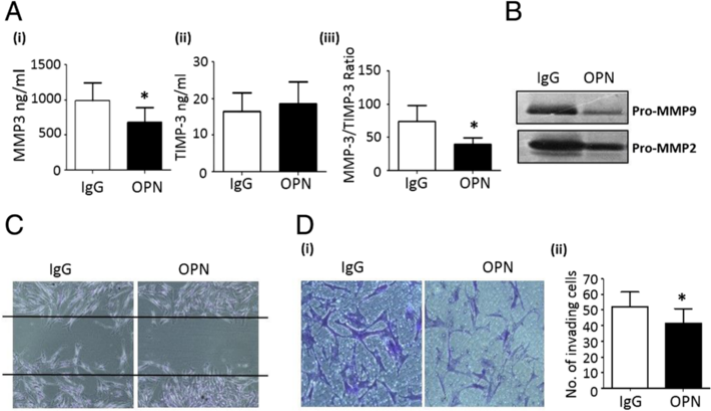
**a** Quantification of RA synovial tissue spontaneous secretion of MMP-3 (*i*), TIMP-3 (*ii*) and the MMP-3/TIMP-3 ratio (*iii*) following culture with OPN301 (1 μg/ml) or IgG control (1 μg/ml) (*n* = 4). **b** Decreased MMP-2 and MMP-9 expression in response to culture with OPN301 (1 μg/ml) compared to anti-IgG control antibody (1 μg/ml) in RA synovial culture supernatants as assessed by gelatin zymography (*n* = 3) (**c**). Representative photomicrograph demonstrating inhibition of RASFC migration in response to conditioned media from ex vivo RA synovial tissue treated with OPN301 (1 μg/ml) compared to IgG control (1 μg/ml) (*n* = 3) (**d**). Representative photomicrographs showing OPN301-conditioned media (1 μg/ml) inhibits RASFC invasion compared to IgG-conditioned media (*i*). Bar graph quantifying RASFC invasion (*ii*) (*n* = 4). Data is represented as mean ± SEM, ^*^ *p* <0.05, significantly different from control. *IgG* immunoglobulin G, *MMP* matrix metalloproteinase, *RA* rheumatoid arthritis, *RASFC* rheumatoid arthritis synovial fibroblast cells, *TIMP* tissue inhibitor of metalloproteinase

To further examine the therapeutic potential of blocking these pathways we assessed the effect of an anti-TLR2 antibody OPN301 in the ex vivo RA synovial explant model. OPN301 significantly reduced spontaneous secretion of MMP-3 from the RA explants compared to IgG control (*p* <0.05) (Fig. 6a (i)), resulting in a significant reduction in the MMP-3:TIMP-3 ratio (Fig. 6a (iii)) (*p* <0.05), thus favouring an MMP/TIMP balance against joint destruction. This was also reflected in the expression of MMP-2 and MMP-9 both of which were inhibited in the presence of OPN301 (Fig. 6b, lane 2) compared to IgG control (Fig. 6b, lane one). OPN301 reduced MMP-1 secretion from explants but this did not reach significance (Figure S1C in Additional file 1). RASFC wounded monolayers were cultured with conditioned media from RA synovial explants treated with OPN301 or IgG control. OPN301-conditioned media inhibited RASFC cell migration across the wound, in contrast to those cultured with IgG control-conditioned media, which had no inhibitory effect (Fig. 6c). Finally, Fig. 6d demonstrates OPN301-conditioned media also inhibited RASFC invasion through a matrigel compared to IgG control. Figure 6d (ii) shows a representative bar graph demonstrating a decreased number of invading RASFC following incubation with OPN301-conditioned media.

## Discussion

RA is a chronic inflammatory disease characterised by increased angiogenesis, cell migration and synovial hyperplasia. TLR2 has been implicated in the pathogenesis of RA, however, the functional mechanisms and signalling pathways mediating its effect on synovial migration and invasion have not been extensively investigated. In this study, we demonstrate that TLR2 activation induces RASFC and HMVEC cell migration, invasion, RA synovial explant outgrowths, in addition to an increase in MMP production. Furthermore, we show that TLR2-induced migration/invasion is partly mediated by β1-integrin and downstream alterations in cytoskeletal dynamics and Rac1 activation. Finally, using an ex vivo RA synovial explant model and RASFC, we demonstrated that blockade of TLR2 significantly inhibited MMP secretion, RASFC migration and invasion.

There is increasing evidence that TLR2 activation plays a critical role in the pathogenesis of RA [6, 26, 27]. Previous studies have demonstrated increased expression of TLR2 in synovial cells and tissue, [4, 5] and shown that TLR2 blockade significantly inhibited spontaneous secretion of pro-inflammatory cytokines from RA synovial explant cultures, an effect similar to that of tumour necrosis factor alpha (TNF-α) inhibitors [22]. In this study, we demonstrate that TLR2 induces RASFC, HMVEC migration and invasion processes, and induces synovial RASFC outgrowth, indicative of the invasive process in RA. Supporting our data, previous studies have demonstrated that TLR2 can induce angiogenic factors and chemokines such as vascular endothelial growth factor (VEGF) and interleukin 8 (IL-8)-promoting cell migrational and angiogenic processes in fibroblasts, chondrocytes, corneal epithelial cells and THP-1 macrophages [28, 29]. Granulocyte-macrophage colony-stimulating factor (GM-CSF) mediates the effect of TLR2/6 agonist, macrophage-activating lipopeptide of 2 kDa (MALP-2) on angiogenic tube formation and leucocytes migration in vivo and in vitro [30]. Furthermore in RASFC, PG activation induces intercellular adhesion molecule (ICAM)-1, interleukin 6 (IL-6) and IL-8 [31]. In addition, Saber et al. 2011, demonstrated in RASFC and RA explant cultures that TLR2 activation induces angiogenic tube formation, adhesion molecule expression and growth factor expression, an effect that was in part mediated through the angiopoietin 2 (Ang2)/Tie-2 pathway [11].

The induction of MMP-3 by TLR2 in RASFC is consistent with a study by Yokota et al. showing TLR2 can synergise with NOD1 in the induction of MMP-3 in RASFC [9]. In addition, a recent study demonstrated that microRNA-19a/b can regulate MMP-3 secretion through activation of TLR2 [10]. Previous studies have shown that MMP-1 and MMP-3 correlate with erosive progression in RA [32]. In addition, studies have shown an association between MMP-3, cartilage neoepitopes, and serum amyloid A (SAA) [33] and demonstrated that MMP-3 levels at baseline predict radiographic progression following TNF-α inhibitor (TNFi) therapy [32]. Furthermore, in human periodontal fibroblasts, TLR2 activation can induce MMP-1 and MMP-3 via p38, JNK and NF-ĸB [34].

We also demonstrated a significant shift in both the MMP-3/TIMP-3 and MMP-1/TIMP-3 ratios suggesting TLR2 activation in the RA joint favours destructive pathways. TLR2 activation inhibited TIMP-3 in RA synovial explants but not RASFC. The expression of TIMP-3 in RA synovial tissue lysates has been previously reported, however, studies did not identify the cellular source of TIMP-3 and the majority of studies in RA only focus on the expression of TIMP-3 in RASFC. However, studies in different disease states and mouse models have demonstrated expression of TIMP-3 in endothelial cells [35], chondrocytes [36] and macrophages [37]. As endothelial cells and macrophages are two prominent cell types in the RA synovium, this suggests that they may be a potential source of the TIMP-3 secreted from explants in response to TLR2 activation.

To further probe the precise mechanisms involved in mediating TLR2-induced synovial invasion, we investigated the role of the β1-integrin signalling pathway. We demonstrated that TLR2 activation preferentially induced β1-integrin binding with no effect observed for β2, β3, β4, β6, αVβ5 or α5β1. Further downstream, we showed that Pam3CSK4-induced Rac1 activation, which is critical for cell actin assembly at the cell periphery promoting cell movement. The effect of TLR2 on β1-integrin and Rac1 was parallelled by alteration in cytoskeletal dynamics where a dramatic effect on the cytoskeletal integrity was observed in both RASFC and HMVEC, consistent with the localised expression of β1-integrin in RA synovial tissue. Finally, we demonstrated that β1-integrin mediated TLR2 function in RASFC and HMVEC, by showing that TLR2-induced migrational and invasive processes were inhibited by neutralising antibodies against β1-integrin.

This is the first study to demonstrate that the β1-integrin pathways specifically mediates TLR2-induced invasion in the inflamed joint, with minimal effect observed for other β-integrins. This is consistent with previous studies showing β-integrin expression on RASFC is associated with their enhanced binding to extracellular matrix (ECM) [38]. The β1-integrin receptor plays a key role in the attachment of RASFC to ECM components and the subsequent invasion of cartilage [18, 38] and can induce Rho GTPases, specifically Rac1 in chondrocytes and murine macrophages [39, 40]. Integrin receptors, p-focal adhesion kinase (FAK) and p-paxillin are significantly increased in RA synovial tissue [41]. β1-integrin is known to be involved in angiogenesis and in the response of T cells to fibronectin-induced chemotactic signals [39, 42]. While little is known about the cross-talk between TLR2 and integrin-FAK-mediated pathways in the RA synovium, previous studies have reported that TLR2 mediates macrophage cytoskeletal rearrangement, promoting macrophage spreading and polarisation, a process required during extravasation and migration [43]. Furthermore, studies have also reported that TLR2 activation induces angiogenesis and EC migration in response to oxidative stress via Rac1 [44, 45] and TLR2(−/−) and MyD88(−/−) macrophages have impaired Rac1 and PI3K function [46].

The mechanisms by which TLR2 mediates β1-integrin action remains unclear. β1-integrin is a transmembrane receptor that is a bridge for cell–cell and cell–ECM interactions, which in turn activates the cytoskeletal dynamics. Ligands for β1-integrin include fibronectin, vitronectin, collagen, and laminin. While no studies in RA cells have shown a direct link between TLR2 and these ligands, a few studies in other disease cells have shown that TLR2 and 4 can regulate the expression of different ECM proteins. Vitronectin and fibronectin have been shown to be essential for TLR2-mediated processes in vitro, both of which are known to be induced in RA [47–51]. Furthermore, while no studies have yet shown a direct link between TLR2 and β1-integrin, we and others have shown that TLR2 is a functional receptor for acute-phase (A)-SAA, which is known to induce cell migration through integrin–cytoskeletal-mediated pathways in the joint [33, 52]. Previous studies have also shown interactions between A-SAA and ECM glycosaminoglycans, and with laminin and vitronectin-induced adhesion and migration in a β1-integrin-dependent manner [53, 54].

In this study, β1-integrin blockade had no effect on MMP-3 expression in RASFC. β1-integrin blockade is required to block MMP-2 activation induced by collagen I (Col I) [55]. Other studies have demonstrated in mice lacking α2β1 integrin, inhibition of MMP-3 expression in RASFC and cartilage degradation [18]. MMP can in turn regulate integrins, with studies showing the regulatory action of MMP-13 on α11β1-dependent matrix remodelling in fibroblasts [56]. However, a study by Ozeki et al., demonstrated complimentary but independent action of α2 integrin and MMP-3-mediated signalling cascades in driving mouse embryonic stem cell differentiation into odontoblast-like cells [57]. Interactions between integrins and MMP in the regulation of matrix remodelling thus may be complimentary and bi-directional with differential sensitivity and activation mechanisms in different cell types or tissues [58].

Finally, we used an ex vivo RA synovial tissue (ST) explant model to investigate the role of TLR2 blockade on RA synovial invasion. This model maintains the cell–cell contact of the complex mix of different cell types whose interaction contributes to the pro-inflammatory environment in the RA joint. RA synovial explants spontaneously release key pro-inflammatory mediators and therefore are ideal for examining potential therapeutic molecules. We demonstrated that OPN301 inhibited spontaneous secretion of MMP-3, MMP-2, and MMP-9 and inhibited the MMP3/TIMP3 ratio in favour of decreased cartilage degradation. We also demonstrated that OPN301-conditioned media inhibited RASFC migration and invasion, suggesting OPN301 inhibited spontaneous release of soluble mediators that are involved in migratory and invasive mechanisms in the RA joint. When RASFC were cultured with OPN301 and IgG alone no inhibitory effect on RASFC migration in response to OPN301 was observed (Figure S1D in Additional file 1). This further suggests endogenous TLR2 ligands are present in the inflamed joint. However, as OPN301 had no direct effect on RASFC migration, ligands may be secreted by other cells in RA synovial tissue. While no ligand has been defined, the existence of a ligand is supported by studies which showed that macrophages were activated in a MyD88 and Mal-dependent manner when cultured with conditioned media from RA synovial explants [8]. Several potential ligands have been suggested such as fibronectin fragments, heat shock protein 70, hyaluronidase oligosaccharides, high mobility group protein 1 (HMBG-1) and glycoprotein 96 (GP96) all of which have been identified in the RA joint [59–63]. These studies support our data suggesting the presence of TLR2 ligands actively secreted from RA ex vivo synovial tissue.

## Conclusions

In conclusion, we demonstrate that activation of TLR2 induces RASFC migration, invasion and extracellular matrix breakdown, through induction of MMPs. We show that TLR2-induced migration/invasion is partly mediated by β1-integrin and downstream alterations in cytoskeletal dynamics. Finally, using an ex vivo RA synovial explant model, we demonstrate that TLR2 blockade significantly inhibits MMP expression and migration, suggesting the presence of an endogenous ligand. This study further supports that hypothesis that TLR2-induced signalling pathways are involved in the pathogenesis of RA.

## Additional file

Additional file 1: Figure S1. (A) Representative photomicrographs showing anti-β1-integrin (10 μg/ml) inhibits Pam3CSK4 (1 μg/ml)-induced HMVEC invasion (i). Bar graph quantifying HMVEC invasion (ii) (*n* = 4). (B) Densitometry quantification of RASFC Pam3CSK4 (1 μg/ml)-induced active Rac1 inhibited in the presence of anti-β1 (10 μg/ml). Data is expressed as fold change compared to β-actin control (*n* = 1) (C) Quantification of RA synovial tissue spontaneous secretion of MMP-1 following culture with OPN301 (1 μg/ml) or IgG control (1 μg/ml) (*n* = 4). (D) Representative photomicrograph demonstrating no change in RASFC migration in OPN301 (1 μg/ml)-treated cells compared to IgG control (1 μg/ml) (*n* = 3).

## Abbreviations

Ang-2: angiopoietin 2
A-SAA: acute-phase serum amyloid A
Col I: collagen I
EBM: endothelial cell basal medium
ECM: extracellular matrix
EGM: endothelial cell growth medium
ELISA: enzyme-linked immunosorbent assay
FAK: focal adhesion kinase
FCS: fetal calf serum
GM-CSF: granulocyte-macrophage colony-stimulating factor
GP96: glycoprotein 96
GTPases: guanosine triphosphates
HMBG-1: high mobility group protein 1
HMVEC: human microvascular endothelial cells
ICAM: intercellular adhesion molecule
IgG: immunoglobulin G
IL-6: interleukin 6
IL-8: interleukin 8
MALP-2: macrophage-activating lipopeptide of 2 kDa
MMP: matrix metalloproteinase
MyD88: myeloid differentiation primary response gene 88
NOD1: nucleotide-binding oligomerisation domain-containing protein 1
PBS: phosphate-buffered saline
PG: peptidoglycan
RA: rheumatoid arthritis
RASFC: rheumatoid arthritis synovial fibroblast cells
SCW: streptococcal cell wall
SM: synovial membrane
ST: synovial tissue
SVUH: St. Vincent’s University Hospital
TIMP: tissue inhibitor of metalloproteinase
TLR: Toll-like receptor
TNFi: tumour necrosis factor inhibitor
TNF-α: tumour necrosis factor α
VEGF: vascular endothelial growth factor

## References

[1] Muller-Ladner U, Kriegsmann J, Franklin BN, Matsumoto S, Geiler T, Gay RE, et al. Synovial fibroblasts of patients with rheumatoid arthritis attach to and invade normal human cartilage when engrafted into SCID mice. Am J Pathol. 1996;1607–15. PMC1865262 8909250

[2] Lefèvre S, Knedla A, Tennie C, Kampmann A, Wunrau C, Dinser R (2009). Synovial fibroblasts spread rheumatoid arthritis to unaffected joints. Nat Med.

[3] Seemayer CA, Kuchen S, Kuenzler P, Rihosková V, Rethage J, Aicher WK (2003). Cartilage destruction mediated by synovial fibroblasts does not depend on proliferation in rheumatoid arthritis. Am J Pathol.

[4] Seibl R, Birchler T, Loeliger S, Hossle JP, Gay RE, Saurenmann T (2003). Expression and regulation of Toll-like receptor 2 in rheumatoid arthritis synovium. Am J Pathol.

[5] Iwahashi M, Yamamura M, Aita T, Okamoto A, Ueno A, Ogawa N (2004). Expression of Toll-like receptor 2 on CD16+ blood monocytes and synovial tissue macrophages in rheumatoid arthritis. Arthritis Rheum.

[6] Schrijver IA, Melief MJ, Tak PP, Hazenberg MP, Laman JD (2000). Antigen-presenting cells containing bacterial peptidoglycan in synovial tissues of rheumatoid arthritis patients coexpress costimulatory molecules and cytokines. Arthritis Rheum.

[7] Brentano F, Kyburz D, Schorr O, Gay R, Gay S (2005). The role of Toll-like receptor signalling in the pathogenesis of arthritis. Cell Immunol.

[8] Sacre SM, Andreakos E, Kiriakidis S, Amjadi P, Lundberg A, Giddins G (2007). The Toll-like receptor adaptor proteins MyD88 and Mal/TIRAP contribute to the inflammatory and destructive processes in a human model of rheumatoid arthritis. Am J Pathol.

[9] Yokota K, Miyazaki T, Hemmatazad H, Gay RE, Kolling C, Fearon U (2012). The pattern-recognition receptor nucleotide-binding oligomerization domain-containing protein 1 promotes production of inflammatory mediators in rheumatoid arthritis synovial fibroblasts. Arthritis Rheum.

[10] Philippe L, Alsaleh G, Suffert G, Meyer A, Georgel P, Sibilia J (2012). TLR2 expression is regulated by microRNA miR-19 in rheumatoid fibroblast-like synoviocytes. J Immunol.

[11] Saber T, Veale DJ, Balogh E, McCormick J, NicAnUltaigh S, Connolly M (2011). Toll-like receptor 2 induced angiogenesis and invasion is mediated through the Tie2 signalling pathway in rheumatoid arthritis. PLoS One.

[12] Brakebusch C, Bouvard D, Stanchi F, Sakai T, Fässler R (2002). Integrins in invasive growth. J Clin Invest.

[13] Nobes CD, Hall A (1995). Rho, rac, and cdc42 GTPases regulate the assembly of multimolecular focal complexes associated with actin stress fibers, lamellipodia, and filopodia. Cell.

[14] Burridge K, Wennerberg K. Rho and Rac take center stage. Cell. 2004;167–179. 10.1016/s0092-8674(04)00003-0 14744429

[15] Bayless KJ, Davis GE (2001). Identification of dual α4β1 integrin binding sites within a 38 amino acid domain in the N-terminal thrombin fragment of human osteopontin. J Biol Chem.

[16] Hoberg M, Rudert M, Pap T, Klein G, Gay S, Aicher WK (2007). Attachment to laminin-111 facilitates transforming growth factor beta-induced expression of matrix metalloproteinase-3 in synovial fibroblasts. Ann Rheum Dis.

[17] Zeisel MB, Druet VA, Wachsmann D, Sibilia J (2005). MMP-3 expression and release by rheumatoid arthritis fibroblast-like synoviocytes induced with a bacterial ligand of integrin alpha5beta1. Arthritis Res Ther.

[18] Peters MA, Wendholt D, Strietholt S, Frank S, Pundt N, Korb A (2012). The loss of integrin α2β1 suppresses joint inflammation and cartilage destruction in mouse models of rheumatoid arthritis. Arthritis Rheum.

[19] Arnett FC, Edworthy SM, Bloch DA, McShane DJ, Fries JF, Cooper NS (1988). The American Rheumatism Association 1987 revised criteria for the classification of rheumatoid arthritis. Arthritis Rheum.

[20] Bain GI, Roth JH (1995). The role of arthroscopy in arthritis. “Ectomy” procedures. Hand Clin.

[21] Youssef PP, Kraan M, Breedveld F, Bresnihan B, Cassidy N, Cunnane G (1998). Quantitative microscopic analysis of inflammation in rheumatoid arthritis synovial membrane samples selected at arthroscopy compared with samples obtained blindly by needle biopsy. Arthritis Rheum.

[22] Ultaigh SN, Saber TP, McCormick J, Connolly M, Dellacasagrande J, Keogh B (2011). Blockade of Toll-like receptor 2 prevents spontaneous cytokine release from rheumatoid arthritis ex vivo synovial explant cultures. Arthritis Res Ther.

[23] Cox RF, Hernandez-Santana A, Ramdass S, McMahon G, Harmey JH, Morgan MP (2012). Microcalcifications in breast cancer: novel insights into the molecular mechanism and functional consequence of mammary mineralisation. Br J Cancer.

[24] Connolly M, Veale DJ, Fearon U (2011). Acute serum amyloid A regulates cytoskeletal rearrangement, cell matrix interactions and promotes cell migration in rheumatoid arthritis. Ann Rheum Dis.

[25] Kleiner DE, Stetler-Stevenson WG (1994). Quantitative zymography: detection of picogram quantities of gelatinases. Anal Biochem.

[26] Drexler SK, Sacre SM, Foxwell BM (2006). Toll-like receptors: a new target in rheumatoid arthritis?. Expert Rev Clin Immunol.

[27] O’Neill LAJ, Bryant CE, Doyle SL (2009). Therapeutic targeting of Toll-like receptors for infectious and inflammatory diseases and cancer. Pharmacol Rev.

[28] Varoga D, Paulsen F, Mentlein R, Fay J, Kurz B, Schütz R (2006). TLR-2-mediated induction of vascular endothelial growth factor (VEGF) in cartilage in septic joint disease. J Pathol.

[29] Monaco C, Gregan SM, Navin TJ, Foxwell BMJ, Davies AH, Feldmann M (2009). Toll-like receptor-2 mediates inflammation and matrix degradation in human atherosclerosis. Circulation.

[30] Grote K, Schuett H, Salguero G, Grothusen C, Jagielska J, Drexler H (2010). Toll-like receptor 2/6 stimulation promotes angiogenesis via GM-CSF as a potential strategy for immune defense and tissue regeneration. Blood.

[31] Kyburz D, Rethage J, Seibl R, Lauener R, Gay RE, Carson DA (2003). Bacterial peptidoglycans but not CpG oligodeoxynucleotides activate synovial fibroblasts by toll-like receptor signaling. Arthritis Rheum.

[32] Green MJ, Gough AKS, Devlin J, Smith J, Astin P, Taylor D (2003). Serum MMP-3 and MMP-1 and progression of joint damage in early rheumatoid arthritis. Rheumatology.

[33] Connolly M, Mullan RH, McCormick J, Matthews C, Sullivan O, Kennedy A (2012). Acute-phase serum amyloid A regulates tumor necrosis factor α and matrix turnover and predicts disease progression in patients with inflammatory arthritis before and after biologic therapy. Arthritis Rheum.

[34] Lisboa RA, Andrade MV, Cunha-Melo JR (2013). Toll-like receptor activation and mechanical force stimulation promote the secretion of matrix metalloproteinases 1, 3 and 10 of human periodontal fibroblasts via p38, JNK and NF-kB. Arch Oral Biol.

[35] Qi JH, Anand-Apte B (2015). Tissue inhibitor of metalloproteinase-3 (TIMP3) promotes endothelial apoptosis via a caspase-independent mechanism. Apoptosis.

[36] Jackson MT, Moradi B, Smith MM, Jackson CJ, Little CB (2014). Activation of matrix metalloproteinases 2, 9, and 13 by activated protein C in human osteoarthritic cartilage chondrocytes. Arthritis Rheum.

[37] Gill SE, Gharib SA, Bench EM, Sussman SW, Wang RT, Rims C (2013). Tissue inhibitor of metalloproteinases-3 moderates the proinflammatory status of macrophages. Am J Respir Cell Mol Biol.

[38] Rinaldi N, Schwarz-Eywill M, Weis D, Leppelmann-Jansen P, Lukoschek M, Keilholz U (1997). Increased expression of integrins on fibroblast-like synoviocytes from rheumatoid arthritis in vitro correlates with enhanced binding to extracellular matrix proteins. Ann Rheum Dis.

[39] Liu-Bryan R, Pritzker K, Firestein GS, Terkeltaub R (2005). TLR2 signaling in chondrocytes drives calcium pyrophosphate dihydrate and monosodium urate crystal-induced nitric oxide generation. J Immunol.

[40] Chen BC, Chang YS, Kang JC, Hsu MJ, Sheu JR, Chen TL (2004). Peptidoglycan induces nuclear factor-kappa B activation and cyclooxygenase-2 expression via Ras, Raf-1, and ERK in RAW 264.7 macrophages. J Biol Chem.

[41] Shahrara S, Castro-Rueda HP, Haines GK, Koch AE (2007). Differential expression of the FAK family kinases in rheumatoid arthritis and osteoarthritis synovial tissues. Arthritis Res Ther.

[42] Hauzenberger D, Klominek J, Sundqvist KG (1994). Functional specialization of fibronectin-binding beta 1-integrins in T lymphocyte migration. J Immunol.

[43] Lasunskaia EB, Campos MNN, de Andrade MRM, Damatta RA, Kipnis TL, Einicker-Lamas M (2006). Mycobacteria directly induce cytoskeletal rearrangements for macrophage spreading and polarization through TLR2-dependent PI3K signaling. J Leukoc Biol.

[44] West XZ, Malinin NL, Merkulova AA, Tischenko M, Kerr BA, Borden EC (2010). Oxidative stress induces angiogenesis by activating TLR2 with novel endogenous ligands. Nature.

[45] Lee IT, Lee CW, Tung WH, Wang SW, Lin CC, Shu JC (2010). Cooperation of TLR2 with MyD88, PI3K, and Rac1 in lipoteichoic acid-induced cPLA2/COX-2-dependent airway inflammatory responses. Am J Pathol.

[46] Shen Y, Kawamura I, Nomura T, Tsuchiya K, Hara H, Dewamitta SR (2010). TLR2-MyD88-dependent PI3K and Rac1 activation facilitates the phagocytosis of Listeria monocytogenes by murine macrophages. Infect Immun.

[47] Liu C-Y, Xu J-Y, Shi X-Y, Huang W, Ruan T-Y, Xie P (2013). M2-polarized tumor-associated macrophages promoted epithelial-mesenchymal transition in pancreatic cancer cells, partially through TLR4/IL-10 signaling pathway. Lab Invest.

[48] Killeen SD, Wang JH, Andrews EJ, Redmond HP (2009). Bacterial endotoxin enhances colorectal cancer cell adhesion and invasion through TLR-4 and NF-kappaB-dependent activation of the urokinase plasminogen activator system. Br J Cancer.

[49] Kulka M, Metcalfe DD (2006). TLR3 activation inhibits human mast cell attachment to fibronectin and vitronectin. Mol Immunol.

[50] Tian YC, Hung CC, Li YJ, Chen YC, Chang MY, Yen TH (2011). Leptospira santorosai Serovar Shermani detergent extract induces an increase in fibronectin production through a Toll-like receptor 2-mediated pathway. Infect Immun.

[51] Gerold G, Ajaj KA, Bienert M, Laws HJ, Zychlinsky A, de Diego JL (2008). A Toll-like receptor 2-integrin beta3 complex senses bacterial lipopeptides via vitronectin. Nat Immunol.

[52] Cheng N, He R, Tian J, Ye PP, Ye RD (2008). Cutting edge: TLR2 is a functional receptor for acute-phase serum amyloid A. J Immunol.

[53] McCubbin WD, Kay CM, Narindrasorasak S, Kisilevsky R (1988). Circular-dichroism studies on two murine serum amyloid A proteins. Biochem J.

[54] Preciado-Patt L, Hershkoviz R, Fridkin M, Lider O (1996). Serum amyloid A binds specific extracellular matrix glycoproteins and induces the adhesion of resting CD4+ T cells. J Immunol.

[55] Borrirukwanit K, Pavasant P, Blick T, Lafleur MA, Thompson EW (2014). High threshold of β1 integrin inhibition required to block collagen I-induced membrane type-1 matrix metalloproteinase (MT1-MMP) activation of matrix metalloproteinase 2 (MMP-2). Cancer Cell Int.

[56] Barczyk MM, Lu N, Popova SN, Bolstad AI, Gullberg D (2013). α11β1 integrin-mediated MMP-13-dependent collagen lattice contraction by fibroblasts: Evidence for integrin-coordinated collagen proteolysis. J Cell Physiol.

[57] Ozeki N, Kawai R, Hase N, Hiyama T, Yamaguchi H, Kondo A (2015). α2 integrin, extracellular matrix metalloproteinase inducer, and matrix metalloproteinase-3 act sequentially to induce differentiation of mouse embryonic stem cells into odontoblast-like cells. Exp Cell Res.

[58] Weber C, Kitayama J, Springer TA (1996). Differential regulation of beta 1 and beta 2 integrin avidity by chemoattractants in eosinophils. Proc Natl Acad Sci U S A.

[59] Schett G, Redlich K, Xu Q, Bizan P, Gröger M, Tohidast-akrad M (1998). Enhanced expression of heat shock protein 70 (hsp70) and heat shock factor 1 (HSF1) Activation in rheumatoid arthritis synovial tissue differential regulation of hsp70 expression and HSF1 activation in synovial fibroblasts by proinflammatory cytokines, shear stress, and antiinflammatory drugs. J Clin Invest.

[60] Huang QQ, Sobkoviak R, Jockheck-Clark AR, Shi B, Mandelin AM, Tak PP (2009). Heat shock protein 96 is elevated in rheumatoid arthritis and activates macrophages primarily via TLR2 signaling. J Immunol.

[61] Scott DL, Delamere JP, Walton KW (1981). The distribution of fibronectin in the pannus in rheumatoid arthritis. Br J Exp Pathol.

[62] Taniguchi N, Kawahara K, Yone K, Hashiguchi T, Yamakuchi M, Goto M (2003). High mobility group box chromosomal protein 1 plays a role in the pathogenesis of rheumatoid arthritis as a novel cytokine. Arthritis Rheum.

[63] Yu D, Rumore PM, Liu Q, Steinman CR (1997). Soluble oligonucleosomal complexes in synovial fluid from inflamed joints. Arthritis Rheum.

