# Supplementary figures and images for: Toll-like receptor 2 (TLR2) induces migration and invasive mechanisms in rheumatoid arthritis
Source: Arthritis Res Ther. 2015 Jun 9;17(1):153. doi: 10.1186/s13075-015-0664-8 (PMC4495696; doi:10.1186/s13075-015-0664-8)

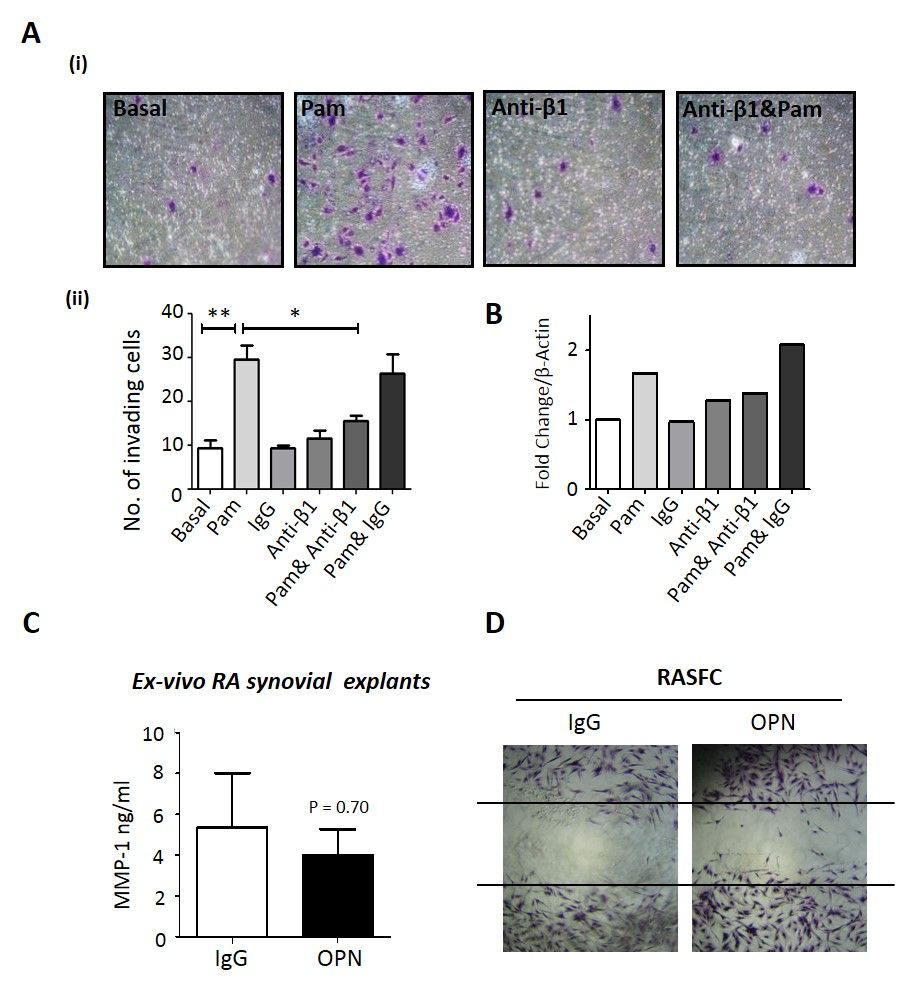

Supplement: Additional file 1: Figure S1. — (A) Representative photomicrographs showing anti-β1-integrin (10 μg/ml) inhibits Pam3CSK4 (1 μg/ml)-induced HMVEC invasion (i). Bar graph quantifying HMVEC invasion (ii) (n = 4). (B) Densitometry quantification of RASFC Pam3CSK4 (1 μg/ml)-induced active Rac1 inhibited in the presence of anti-β1 (10 μg/ml). Data is expressed as fold change compared to β-actin control (n = 1) (C) Quantification of RA synovial tissue spontaneous secretion of MMP-1 following culture with OPN301 (1 μg/ml) or IgG control (1 μg/ml) (n = 4). (D) Representative photomicrograph demonstrating no change in RASFC migration in OPN301 (1 μg/ml)-treated cells compared to IgG control (1 μg/ml) (n = 3). [file 13075_2015_664_MOESM1_ESM.jpeg]
